# Blood pressure control of hypertensive patients followed in a high complexity clinic and associated variables

**DOI:** 10.1590/2175-8239-JBN-2020-0133

**Published:** 2021-02-15

**Authors:** Juliana Chaves Coelho, Mayra Cristina da Luz Pádua Guimarães, Cassia Lima de Campos, Carime Farah Florido, Giovanio Vieira da Silva, Angela Maria Geraldo Pierin

**Affiliations:** 1 Universidade de São Paulo, Escola de Enfermagem, São Paulo, SP, Brasil.; 2 Hospital Sírio-Libanês, São Paulo, SP, Brasil.; 3 Universidade de São Paulo, Faculdade de Medicina, Hospital das Clínicas, São Paulo, SP, Brasil.

**Keywords:** Hypertension, Control, Drug Therapy, Hipertensão, Controle, Tratamento Farmacológico

## Abstract

**Introduction:**

Arterial hypertension is a disease that has a high impact on cardiovascular mortality and morbidity; however, it is still insufficiently controlled.

**Objectives:**

To assess hypertension control in patients seen at a specialized clinic and to identify associated variables.

**Method:**

Cross-sectional study involving the analysis of medical records from 782 patients treated in a highly complex outpatient clinic. Inclusion criteria: age ≥18 years, diagnosed with hypertension, in treatment ≥6 months. Patients with secondary hypertension (104) and incomplete data (64) were excluded. The main outcome was blood pressure control (systolic <140 and diastolic <90 mmHg). The independent variables studied were: sociodemographic and clinical characteristics (use of drugs, comorbidities and laboratory tests). Pearson's χ2 tests, Fisher's test, Student's t and Wilcoxon-Mann-Whitney tests were performed in the bivariate analysis and logistic regression in the multiple analyses, adopting p≤0.05.

**Results:**

The prevalence of hypertensive control was 51.1%. It was associated with a lack of control: body mass index (OR = 1.038; 95% CI = 1.008 - 1.071), history of stroke (OR = 0.453; 95% CI = 0.245 - 0.821), left ventricular hypertrophy (OR = 1.765; 95% CI = 1.052 - 3.011), and number of medications (OR = 1.082; 95% CI = 1.033 - 1.136).

**Conclusion:**

About half of the hypertensive patients had their blood pressure controlled; clinical variables and target organ damage were associated with the control.

## Resumo

**Introdução:**

A hipertensão arterial é uma doença com alto impacto na mortalidade e morbidade cardiovascular, contudo ainda demonstra insuficientes taxas de controle.

**Objetivos:**

Avaliar o controle da hipertensão em pacientes atendidos em um ambulatório especializado e identificar variáveis associadas.

**Método:**

Estudo transversal com análise do prontuário de 782 pacientes atendidos em um ambulatório de alta complexidade. Critérios de inclusão: idade ≥ 18 anos e diagnóstico de hipertensão em tratamento ≥ 6 meses. Foram excluídos hipertensão secundária (104) e dados incompletos (64). O desfecho principal foi o controle da pressão arterial (Sistólica < 140 e diastólica < 90 mmHg). As variáveis independentes estudadas foram: características sociodemográficas e clínicas (uso de medicamentos, comorbidades e exames laboratoriais). Realizou-se testes χ2 de Pearson, teste Fisher, t de Student e Wilcoxon-Mann-Whitney na análise bivariada e Regressão Logística na análise múltipla, adotando *p* ≤ 0,05.

**Resultados:**

A prevalência de controle dos hipertensos foi 51,1%. Associou-se à falta de controle: índice de massa corporal (OR = 1,038; IC95% = 1,008 - 1,071), histórico de acidente vascular encefálico (OR = 0,453; IC95% = 0,245 - 0,821) e hipertrofia ventricular esquerda (OR = 1,765; IC95% = 1,052 - 3,011), e número de medicamentos (OR = 1,082; IC95% = 1,033 - 1,136).

**Conclusão:**

Cerca da metade dos hipertensos estava com pressão arterial controlada e variáveis clínicas e lesão em órgão alvo associaram-se ao controle.

## Introduction

Arterial hypertension is one of the diseases that most contributes to cardiovascular complications, with a high impact on mortality and morbidity^1^, in addition to being the main risk factor for global disease burden^2^. The prevalence of hypertension has remained somewhat stable in several countries around the world^3^, reaching about 30% of the population in Brazil^4^. On the other hand, the disease control, despite having had a significant increase over the last decades in many countries, still maintains unsatisfactory, around 50% in the best scenarios^3^^,^ 5 ^,^ 6. Other Brazilian studies point to a control variation in hypertensive patients, from 33.7% to 67.5%^7^^,^ 8, and all these data correspond to patients treated in primary healthcare.

Blood pressure control is the main goal of hypertension treatment and, when achieved, it reduces cardiovascular events^9^. A 10-mmHg drop in systolic blood pressure reduced in about 17% the coronary events, strokes in 27%, and heart failure in 28%^10^. Despite the benefits, achieving half the control of hypertensive patients is still a major challenge. This involves complex aspects, such as drug treatment compliance, which has particularities related to disease chronicity, access to healthcare services and the very biosocial characteristics of hypertensive patients. As a result, many patients with complications from hypertension need additional care, and are often followed by specialized services. In a national systematic review, whose control rates ranged from 10% to 57.6%, only 24.4% of the publications analyzed hypertensive patients seen in secondary care centers^11^.

Thus, national data on hypertension control are centered on primary care. A fact already expected, considering that this is where we concentrate care to hypertensive patients. However, hypertensive patients with greater severity due to target organ injury, associated with comorbidity are seen in specialized services, and there is a lack of data on the control of these patients. Therefore, the present study aimed to assess the prevalence of hypertension control to identify associated variables, in a specialized hypertension care at a tertiary healthcare level.

## Methods

### Population

**Figure 1:**
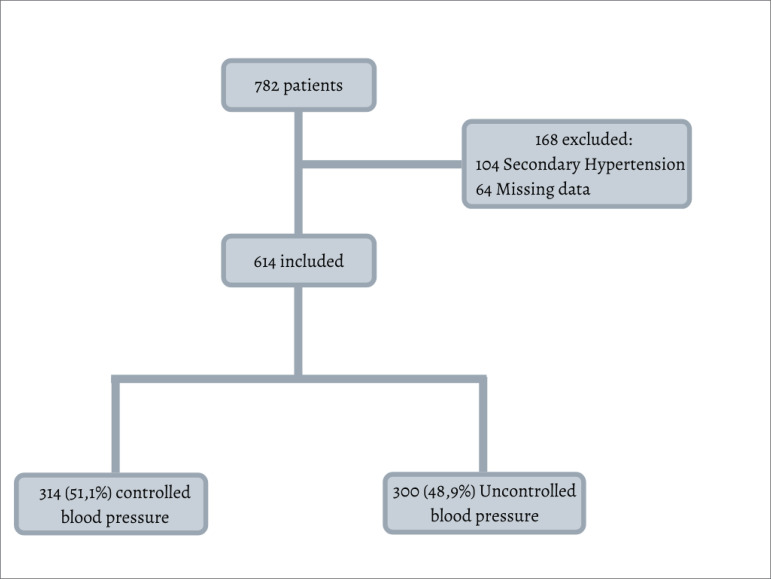
Flowchart of the inclusion and exclusion processes of the hypertensive patients - São Paulo, 2019.

This is a cross-sectional study, carried out with data from the electronic medical records of 782 hypertensive patients. This population was taken from the schedule of medical consultations held in the last nine months at the Hypertension Clinic, in the department of Nephrology, of a Tertiary Teaching Hospital in the city of São Paulo. The outpatient clinic serves approximately 850 highly complex hypertensive patients, referred by primary care for specialized follow-up. The inclusion criteria were age above 18 years old, hypertensive and undergoing treatment for at least six months in the clinic. We had 104 with a diagnosis of secondary hypertension, and 64 being taken out due to insufficient data (Figure 1). Since this is a study using secondary data from electronic medical records, the Informed Consent Form was waived, and it was approved from the ethics committee of the University of São Paulo School of Nursing (Protocol #: 3.519.736 / 2019) and of the ethics committee of the University of São Paulo Medical School University Hospital (Protocol #: 3,617,641/2019).

### Data collection

The data was retrospectively collected from the patients' electronic medical records. The dependent variable was blood pressure control, defined as systolic blood pressure lower than 140 mmHg and diastolic blood pressure lower than 90 mmHg, in at least two of the last three medical appointments. The independent variables analyzed were demographic characteristics including age (defined by date of birth), sex (female or male), race (white, black, brown, mulatto or yellow) and marital status (single, married, cohabiting, separated or widowed). The clinical characteristics evaluated were: weight and height, for calculating the Body Mass Index; history of stroke (medical record of medical diagnosis of hemorrhagic, ischemic or unspecified stroke, in addition to transient cerebral ischemia); coronary insufficiency (a record of medical diagnosis of coronary insufficiency, stable or unstable angina, angina pectoris or acute myocardial infarction); resistant hypertension (medical records of resistant hypertension); chronic kidney disease (estimated glomerular filtration rate, obtained by the MDRD equation < 60 mL/min or recorded in the renal failure diagnosis chart); diabetes (medical diagnosis chart, or two results of fasting blood glucose ≥ 126 mg/dL or glycated hemoglobin ≥ 6.5 mg/dL or medical prescription of a hypoglycemic agent); dyslipidemia (medical diagnosis records or LDL cholesterol fraction > 130 mg/dL or medical prescription for lipid-lowering drugs); and left ventricular hypertrophy (recorded in the medical diagnosis chart, or echocardiogram result with left ventricular mass index > 96 g/m^2^ for females and > 116 g/m^2^ for males). The laboratory exams analyzed were fasting blood glucose, glycated hemoglobin, lipid profile (total cholesterol, LDL fraction, HDL and triglycerides), and glomerular filtration rate using the MDRD equation, urea, creatinine and proteinuria. The drug treatment was assessed using the drug records of the last medical prescription. The history of diseases was identified in the data recorded in the last three consultations. For the evaluation of anthropometric data, laboratory tests and blood pressure, we considered the values measured during the last consultation. Previously trained nurses and graduate students collected the data.

### Data analysis and processing

We used the R software to run the statistical analyzes. For the sorting variables, we used the Pearson's χ2 and Fisher's exact tests; and for the continuous tests, the Student's t-test or Wilcoxon-Mann-Whitney. We set the level of significance at *p*≤0.05. In the logistic regression analysis, variables with *p* < 0.20 were included in the bivariate analysis.

## Results

We had 614 hypertensive patients participating in the study, half of whom (51.1%) had controlled blood pressure. The average follow-up time for patients at the clinic was 5.73 ± 2.72 years.

Table 1 shows the sociodemographic data. Most of the hypertensive patients were white females, about half were married and in their sixth decade of life. Controlled hypertensive patients are different (*p*≤0.05) from uncontrolled ones, because they are younger [61.2 (16.0) vs. 66.4 (13.2) years] and have a higher percentage of black race (37, 1% vs. 62.9%).

**Table 1:** Sociodemographic characteristics of the controlled and uncontrolled hypertensive patients seen at a high-complexity outpatient clinic - São Paulo, SP, 2019

	BP control						
Variables	Yes		No		Total		p value
	n	%	n	%	N	%	
Sex							0.231
Females	198	49.4	203	50.6	401	65.3	
Males	116	54.5	97	45.5	213	34.7	
**Etnia (N = 605)**							**0.003^a^**
White	244	54.3	205	45.7	449	74.2	
White-Black mix	30	40.0	47	60.0	77	12.8	
Black	23	37.1	39	62.9	62	10.2	
Brown	9	81.8	2	18.2	11	1.8	
Yellow	2	33.3	4	66.7	6	1.0	
**Marital status** **(N = 601)**							0.155
Married	155	50.1	154	49.9	309	51.4	
Single	91	58.3	65	41.7	156	26.0	
Separated	26	46.4	30	53.6	56	9.3	
Widow (er)	22	40.0	33	60.0	55	9.1	
Living together	12	48.0	13	52.0	25	4.2	
**Age (years)-** **Mean (SD)**	61.2 (16.0)		66.4 (13.2)		64.2 (14.8)		**< 0.001^b^**

a p - obtained by the Pearson's χ^2^ test;

b Welch's two-sample test.

Concerning a personal history, half of the hypertensive patients had a history of Dyslipidemia, and just over a third had diabetes mellitus, followed by chronic renal failure, obesity and resistant arterial hypertension. There was lower rates of left ventricular hypertrophy, stroke and coronary heart failure. Hypertensive patients had a lipid profile with total cholesterol and triglyceride values in the normal range; the LDL fraction was in the low risk range; and the HDL fraction was within desirable values. Fasting blood glucose was in the inappropriate range, and 78.3% had values ≥ 126 mg/dL. Glycated hemoglobin was in the risk range for developing diabetes: 26.5% of hypertensive patients had values above 6.5 mg/dL. Creatinine and urea were within the normal range. Proteinuria was present in 30% of the hypertensive patients. About the glomerular filtration rate, using the MDRD equation, despite the average with normal value, 32% presented values below 60mL/min. The body mass index remained at the upper limit of the overweight range, and 76.2% were overweight/obese. The systolic pressure value was barely above the control value, but with controlled diastolic pressures. In relation to the uncontrolled, the data of the controlled hypertensive patients were statistically different (*p*≤0.05), because they had less history of diabetes mellitus (43.1% vs 56.9%), obesity (42.1% *vs*. 57.9% ), resistant hypertension (37.6% *vs*. 62.3%) and left ventricular hypertrophy (35.7% *vs*. 64.3%); as well as lower proteinuria (43.5% vs 56.5%), lower mean triglycerides [132.0 (61.1) vs 146.6 (81.2) mg / dL], fasting blood glucose [105, 8 (29.4) vs 114.7 (36.9) mg / dL], glycated hemoglobin [5.9 (1.1) vs 6.3 (1.4)%], creatinine [1.1 (0 , 7) vs 1.2 (1.1) mg / dL], weight [73.2 (15.3) vs 76.6 (16.1) Kg], body mass index [28.5 (5 , 7) vs 30.1 (6.5) kg / m^2^] and higher glomerular filtration rate [69.4 (24.1) vs 66.5 (25.1)]. As for blood pressure values, the controlled hypertensive patients had a mean systolic and diastolic blood pressure levels significantly lower than those of uncontrolled patients (Table 2).

**Table 2:** Comorbidities and laboratory tests of controlled and uncontrolled hypertensive patients seen at a high-complexity outpatient clinic in São Paulo, SP, 2019

	BP Control						
Variables	Yes		No		Total		p value
	n	%	n	%	n	%	
**Personal history**							
Dyslipidemia	158	51.5	149	48.5	307	50.0	0.872
Diabetes mellitus	98	43.1	129	56,9	227	36.9	**0.003^a^**
Chronic renal failure	80	46.0	94	54,0	174	28.3	0.108
Obesity	67	42.1	92	57.9	159	25.9	**0.008^a^**
Resistant hypertension	58	37.6	96	62.3	154	25.1	**< 0.001^a^**
Left ventricular hypertrophy	30	35.7	54	64.3	84	13.7	**0.002^a^**
Stroke	38	60.3	25	39.7	63	10.2	0.124
Coronary insufficiency	28	52.8	25	47.2	53	8.6	0.797
**Lipid profile** (mg/dL) Mean (DP)							
Total cholesterol	177.6 (41.5)		180.3 (39.1)		179.3 (40.3)		0.420
Triglycerides	132.0 (61.1)		146.6 (81.2)		139.2 (72.1)		**0.013^c^**
HDL	53.8 (15.0)		53.7 (16.3)		53.8 (15.6)		0.947
LDL	99.9 (33.6)		101.6 (31.3)		100.8 (32.5)		0.539
**Mean glucose** (SD)							
Fasting glucose (mg/dL)	105.8 (29.4)		114.7 (36.9)		110.2 (33.5)		**0.001^b^**
Glycated hemoglobin (%)	5.9 (1.1)		6.3 (1.4)		6.1 (1.2)		**0.003^c^**
**Renal function**							
Proteinuria	78	43.5	101	56.5	196	30.7	**0.027^a^**
Urea (mg/dL) mean (SD)	41.6 (22.8)		44.1 (23.2)		42.8 (23.0)		0.178
Mean creatinine (mg/dL) (SD)	1.1 (0.7)		1.2 (1.1)		1.1 (0.9)		**0.024^b^**
Glomerular filtration rate (MDRD)- mean (SD)	69.4 (24.1)		66.5 (25.1)		68.0 (24.6)		**0.034^c^**
**Anthropometric characteristics** mean (SD)							
Weight	73.2 (15.3)		76.6 (16.1)		74.8 (16.3)		**0.012^b^**
Height	160.3 (9.8)		159.3 (9.6)		159.8 (9.7)		0.224
Body mass index	28.5 (5.7)		30.1 (6.5)		29.3 (6.2)		**0.001^c^**
**Blood pressure (mmHg)** mean (SD)							
Systolic BP	129.7 (14.5)		155.0 (21.1)		142.1 (22.0)		**< 0.001^b^**
Diastolic BP	73.6 (10.5)		82.6 (15.8)		78.1 (14.1)		**< 0.001^b^**

a p - obtained by the Person's χ^2^ test;

b Welch two-sample test;

c Two-sample t-test.

Regarding drug treatment, 11 patients (1.8%) were not prescribed antihypertensive drugs which was the most frequent medication class among the patients. After antihypertensive drugs, the most prescribed medication class was lipid-lowering agents, with just over half (58.1%), followed by anticoagulants/antiplatelet drugs (44.8%) and antacids (42.3%), and prescribed for slightly less of half the patients. About a third used hypoglycemic agents (32.7%), as well as non-opioid analgesics/muscle relaxants (31.7%). To a lesser extent, they took vitamins and digestive enzymes (24.8%), antidepressants (19.4%), medicines for thyroid treatment (16.1%), opioid analgesics (10.5%) and anti- inflammatory (8.7%).

The data presented in Table 3 show that the average number of drugs on the medical prescription was almost nine drugs for hypertension, of which little more than three corresponded to antihypertensive agents. Only 5.5% of the hypertensive patients had a prescription for only one antihypertensive agent, and the rest were practically divided into two or three, or four or more classes of different antihypertensive drugs. Regarding the prescribed classes of antihypertensive agents, most were diuretics and calcium channel blockers, with hydrochlorothiazide and amlodipine being the most frequent. Beta- blockers and angiotensin II receptor blockers were prescribed for almost half of the hypertensive patients, the most frequent of which were atenolol and losartan. Approximately one third took angiotensin-converting enzyme inhibitors, in which enalapril was the most used. In smaller proportions, they took centrally acting agents, vasodilators and alpha-blockers. Hypertension-controlled patients were statistically different from their uncontrolled counterparts (*p*≤0.05), due to the lower average of medications in general [8.0 (4.2) *vs*. 9.9 (4.0)] and antihypertensive drugs [2.9 (1.3) *vs*. 3.7 (1.2) respectively], less use of four or more antihypertensive drugs (37.0% *vs*. 63.0%, respectively); and lower number of different classes of antihypertensive agents, except for alpha-blockers.

**Table 3:** Drug treatment characteristics of controlled and uncontrolled hypertensive patients seen at the high-complexity outpatient clinic- São Paulo, SP, 2019

	BP control						
Variables	Yes		No		Total		p value
	n	%	n	%	n	%	
**Number of medications** mean (SD)							
	8.0 (4.2)		9.9 (4.0)		8.9 (4.2)		< 0.001
**Number of anti-hypertensive agents (SD)**							
	2.9 (1.3)		3.7 (1.2)		3.3 (1.3)		**< 0.001**
Anti-hypertensive use	28	82.4	6	17.6	34	5.5	**< 0.001**
Two-three anti-hypertensive	173	58.9	121	41.1	294	47.9	
Four or more anti-hypertensive	102	37.1	173	62.9	275	44.9	
**Classes of anti-hypertensive**							
Diuretics	235	43.7	270	56.3	505	64.6	**< 0.001**
Calcium-channel blockers	193	41.3	245	58.7	438	56.0	**< 0.001**
Beta blockers	158	42.4	190	57.6	348	44.5	**0.001**
Angiotensin receptor blockers							
	165	44.1	186	55.9	351	44.9	**0.018**
ACE inhibitors	93	43.4	113	56.6	206	26.3	**0.035**
Central-acting drugs	25	24.5	69	75.5	94	12.0	**< 0.001**
Vasodilators	30	34.8	51	65.2	81	10.4	**0.006**
Alpha-blockers	17	51.5	12	48.5	29	3.7	0.409

^a^ p - obtained by the Pearson's χ^2^ test; ^b^ Welch's two-sample test; ^c^ two-sample t-test.

The multiple regression model showed that the following variables were associated with a lack of control (*p*≤0.05): body mass index; history of stroke and left ventricular hypertrophy; and number of prescription drugs. Having a history of stroke reduced the chance of uncontrolled hypertension by 55%, while the history of left ventricular hypertrophy increased by 76%. With each increase in the body mass index, the chance of non-control increased by 3.8%, and with each medication added to the prescription, the chance of non-control increased by 8.2% (Table 4).

**Table 4:** Logistic regression model: variables associated with the lack of blood pressure control in hypertensive patients seen at the high-complexity outpatient clinic- São Paulo, SP, 2019

Age	Odds Ratio	CI (95%)	p value
**Age**	1.007	0.993 - 1.022	0.302
**Ethnics**			
Brown	0.114	0.008 - 1.180	0.079
White	0.336	0.042 - 2.179	0.253
White-Black mix	0.630	0.075 - 4.300	0.637
Black	0.668	0.079 - 4.595	0.682
**Marital status**			
Single	0.711	0.279 - 1.804	0.470
Married	0.863	0.350 - 2.122	0.746
Separated	1.048	0.369 - 2.976	0.929
Widow (er)	1.145	0.395 - 3.327	0.802
**Body mass index**	1.038	1.008 - 1.071	**0.014**
**Fasting glucose**	1.005	0.999 - 1.010	0.109
**Personal history**			
Stroke	0.453	0.245 - 0.821	**0.010**
Resistant hypertension	1.354	0.887 - 2.071	0.160
Left ventricular hypertrophy	1.765	1.052 - 3.011	**0.034**
**Number of drugs** **prescribed**	1.082	1.033 - 1.136	**0.001**

## Discussion

The results of the present study showed that, despite the complexity of the analyzed hypertensive patients^12^^,^ 13, the prevalence of blood pressure control was 51.1%, which seems to reflect current Brazilian estimates. Data from the Longitudinal Study of Elderly Health, whose participants had a similar average age, and from the First Brazilian Hypertension Registry^6^^)^ showed that control rates were around 50%. The same was reported in a regional study, in which about 45% of patients were controlled^14^. On the other hand, when looking at the results of previous years, the control in Brazil was lower^15^^,^ 16. In addition, these data show hypertensive patients followed, in general, by primary care, in which patients with less severe disease are concentrated. In this sense, such control estimates can be considered unsatisfactory. There are few studies evaluating control in a population similar to the one in the present study, and the fact that many of them present injury to target organs, and other concomitant diseases may represent a complicating factor to reach pressure targets.

Despite the robust evidence^10^ on the impact of a reduction in cardiovascular morbidity and mortality when blood pressure levels are reduced, the failure to effectively control BP and the burden on the health system that the complications of arterial hypertension represent are still major challenges for everyone, including developed countries.

The prevalence of control in the best possible scenarios is only reasonable. Recent data showed poor results concerning control rates in twelve high-income countries: Finland, Ireland, Italy, Japan and Spain had the lowest rates (< 20% in some age groups and sexes), while Canada and Germany had the highest (50% to 58% among women and 48% to 69% among men, respectively)^3^. When compared to these results, the data of the present study stand out in a positive way, although an important gap remains concerning the effective treatment of hypertension.

WeIt was also reported on which factors were associated with blood pressure control. With regard to biosocial data, the black race was more prevalent among the group of uncontrolled hypertensive individuals, as well as older age; however, such variables did not remain in the final logistic regression model. It is widely described in the literature^17^^,^ 18 ^,^ 19, that black ethnicity is related to higher blood pressure levels, when compared to white ethnicity, which may be associated with genetic predisposition; however, unsatisfactory socioeconomic levels are more relevant and are associated with poorer access to health services. In relation to age, some studies suggest a tendency to increase control with increasing age^20^^,^ 21 ^,^ 22, which is not in contrast to what was found in this study, considering the predominance of the age group in the sixth decade. The higher prevalence of arterial hypertension as age increases is related to vascular changes, resulting from endothelial dysfunction, vascular remodeling, increased vascular stiffness and inflammation^23^. Thus, the elderly have an additional challenge in controlling blood pressure.

As for the laboratory profile, significant changes were seen in uncontrolled hypertensive individuals, such as a greater presence of proteinuria and serum creatinine levels, and a lower glomerular filtration rate. The higher level of triglycerides, fasting glycemia and glycated hemoglobin also attracted attention. Although none of them remained in the final model, these characteristics showed the relationship between the lack of pressure control and the occurrence of several other diseases. Such alterations suggest high cardiovascular risk and, even though many are modifiable factors in the prevention of cardiovascular disease^24^, they can still cause problems to clinical management. This is the case with Body Mass Index^25^, which increase was independently associated with the lack of control of hypertensive patients. It is known that the risk of hypertension continually increases with the increase in Body Mass Index, and the opposite is true, since the decrease in weight acts with reductions in blood pressure levels^26^.

Thus, when assessing the presence of other comorbidities, we found that uncontrolled hypertensive patients had higher percentages of diabetes, obesity, resistant hypertension and left ventricular hypertrophy. Diabetes, in cases of hypertension, elevates the patient to the group of highest risk for cardiovascular disorders^27^,which, when added to the uncontrolled pressure levels, can cause a greater probability of changes in target organs.

Left ventricular hypertrophy remained independently associated, representing an installed cardiovascular complication, directly associated with the lack of long-term control. The relationship between pressure control and ventricular hypertrophy can be seen with some results from the SPRINT study, in which intensive blood pressure control in patients without left ventricular hypertrophy at baseline was associated with a 46% lower risk of developing hypertrophy at the end of the study^28^.^( )^In a different way, the history of stroke reduced the chance of not being controlled and the model of multiple analysis remains. Possibly, these findings suggest that left ventricular hypertrophy, being asymptomatic and requiring diagnostic imaging, often does not have an impact on the patient's behavior in the sense of increasing healthcare, unlike what happens with a patient affected by a stroke, often hospitalized, with the risk of developing sequelae and imminent risk of death. In this perspective, stroke is the second leading cause of death in the world and the third most common cause of disability^29^.Therefore, patients who recover from this condition have more stringent goals in controlling blood pressure and the factors that can cause a new injury.

Regarding antihypertensive drug therapy, most of the sample used combinations of two to three drug classes or four or more classes, possibly related to the severity profile of patients, often with the presence of associated diseases, such as diabetes and chronic renal failure. A study with a similar methodology, carried out in primary care, found that 60.5% of uncontrolled hypertensive patients had the prescription of three or more antihypertensive drugs^30^.

The results of the present study showed that the increase in the number of medications increased the chance for poor control, a fact that is already well portrayed in the literature^31^.Possibly, increasing the number of medications that may have an impact on treatment compliance, since it may represent greater numbers of doses, and be influenced by the forgetfulness factor, reflecting in worse control.

Some limitations of the study may be associated with the use of secondary data, as important aspects, such as compliance to treatment, could not be evaluated. Information such as a past of diseases and the presence of resistant hypertension were reported in medical records and could not be confirmed by more precise diagnostic criteria, but it should be noted that the percentages found were similar to laboratory rates and the prescription of corresponding drugs. Thus, we conclude that the studied hypertensive patients had a profile of greater cardiovascular severity, in addition to reasonable blood pressure control.

## Conclusion

The data from the present study indicated that about half of the hypertensive patients had their blood pressure under control. Evaluating the most complex profile of the studied population and similar estimates in developed countries, this data can be considered encouraging. The profile of hypertensive patients outlined can provide essential data to establish strategies aimed at meeting the real needs of hypertensive patients, especially with regards to treatment compliance, which may have an impact on maximizing control and, consequently, modifying the morbidity and mortality profiles of this population.
